# Spectrum of gastrointestinal lesions of neurofibromatosis type 1: a pictorial review

**DOI:** 10.1007/s13244-018-0648-8

**Published:** 2018-09-04

**Authors:** Nada Garrouche, Amel Ben Abdallah, Nadia Arifa, Ibtissem Hasni, Yasser Ben Cheikh, Waad Ben Farhat, Sana Ben Amor, Hela Jemni

**Affiliations:** 1 Radiology Department, Sahloul University Hospital, Sousse, Tunisia; 2 General Surgery Department, Sahloul University Hospital, Sousse, Tunisia; 3 Neurology Department, Sahloul University Hospital, Sousse, Tunisia

**Keywords:** Neurofibromatosis type 1, Recklinghausen disease, Gastrointestinal tract, Neurofibroma, Neuroendocrine tumour, GIST

## Abstract

**Abstract:**

Neurofibromatosis type 1 (NF1) is one of the most common genetic disorders. Gastrointestinal manifestations of NF-1 are seldom thought of in routine clinical practice and might thus be significantly under-recognised. Their heterogeneous spectrum ranges from localised microscopic proliferative lesions to grossly recognizable mass-forming neurofibromas, neuroendocrine and gastrointestinal stromal tumours (GIST). The aim of this study is discussing the imaging evaluation and characterisation of the abdomen lesions in patients with NF1.

**Teaching Points:**

• *Neurofibromatosis type (NF-1) is one of the most common single gene disorders.*

• *Every organ system can be involved and intra-abdominal manifestations are underestimated.*

• *The NF1 abdominal manifestations comprehend five categories of tumours.*

• *Neurogenic tumours including with neurofibromas are the most common type.*

• *Early diagnosis of abdominal manifestations of NF-1 based on imaging patterns is necessary for appropriate treatment to avoid serious organic complications related to tumour mass.*

## Introduction

Neurofibromatosis type 1 (NF1) or Recklinghausen disease is a tumour syndrome caused by alterations in the NF-1 gene [1, 2]. It belongs to a group of disorders referred to as phakomatoses or neurocutaneous syndromes [3, 4], including neurofibromatosis (type 1 and type2 ), tuberous sclerosis (Bourneville-Pringle disease), Von Hippel-Lindau (VHL) disease, Sturge-Weber syndrome (SWS) and many other neurocutaneous diseases (ataxia telangiectasia, incontinentia pigmenti, etc.) [5]. These disorders have selective involvement of tissues of ectodermal origin and are characterised by systemic hamartomas of the eye, brain and sometimes viscera and bones [1, 3, 5].

In contrast to neurofibromatosis type 2, which is considered as the central nervous system neurofibroma, in NF1 other systems can also be affected, including the cardiovascular system, bones and gastrointestinal (GI) tract [6].

NF1 is an autosomal dominant disorder. Prevalence is 1 in 3000 people, with half of patients having a family history and half of cases arising spontaneously [6].

Gastrointestinal system involvement represents 10%–25% of all patients [6, 7] and is seldom thought of in routine clinical practice. The upper intestinal tract is more affected and neurofibromas are the most common type of lesions located mostly in the small intestines.

Since the NF1 abdominal lesions are often asymptomatic and might thus be significantly under-estimated, the aim of this study is to review the gastrointestinal lesions in patients with NF1 and to discuss the imaging features and characteristics of these lesions.

## Overview of NF1 disease

**Fig. 1:**
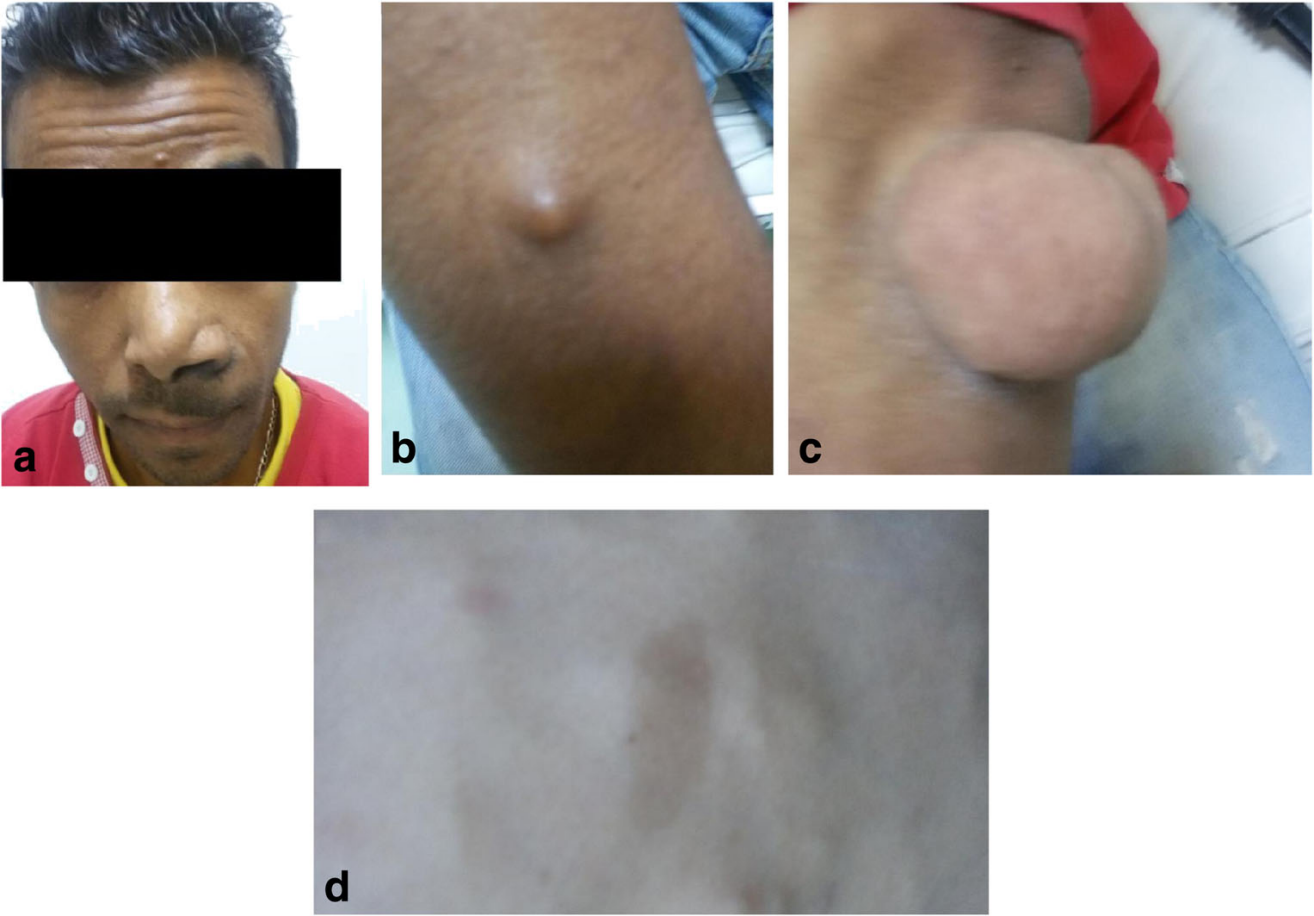
Different cutaneous manifestations of NF1 including: (**a**) plexiform neurofibroma in the face; (**b**, **c**) neurofibromas; (**d**) “café au lait” spots

Many of the clinical and neoplastic manifestations of NF1 are related to age [8]. The most common clinical manifestations are “café au lait” spots, neurofibromas, Lisch nodules (defined as pigmented hamartomatous nodular aggregates of dendritic melanocytes affecting the iris) and axillary or inguinal freckling (increased number of freckles, small circular spots on the skin that are darker than the surrounding skin, because of deposits of melanin) (Fig. 1) [1, 7]. Abdominopelvic involvement in NF1 is primarily extraperitoneal, mostly detected in the abdomino-pelvic wall and lumbosacral plexus [6, 7, 9, 10].

Abdominal neoplasms are frequent in patients with NF1 because of the mutation in the NF1 gene located on chromosome 17q11.2. NF1 neurofibromin [7], a tumour suppressor protein, is involved in the regulation of several cellular signalling pathways responsible for cell proliferation and differentiation [11]. The severity of this disorder ranges from benign (75%) to very aggressive conditions (25%) [7]. The localised and plexiform neurofibromas of the paraspinal and sacral region are the most common abdominal manifestations in NF1 [7, 9].

Visceral abdominal neoplasms occurring in patients with NF1 fall into five categories of neoplasms, presented in Table 1 [7, 12]: neurogenic tumours, neuroendocrine tumours, non-neurogenic gastrointestinal stromal tumours, embryonal tumours and miscellanea. Benign and malignant neoplasms may arise in the abdomen in both paediatric and adult patients with NF1 [12, 13].

**Table 1:** Different type of gastrointestinal tract lesions associated with NF1

True neurogenic neoplasm	Solitary neurofibroma Diffuse or plexiform neurofibroma Gastric schwannoma (1 case reported) Diffuse mucosal/submucosal neurofibromatosis Ganglioneuromatosis Gangliocytic paraganglioma Malignant peripheral nerve sheath tumour (very rare)
Interstitial cell of Cajal lesions	Multifocal clinical gastrointestinal stromal tumours (GISTs) Minute incidental GIST tumourlets (usually non-gastric) Microscopic diffuse or multifocal interstitial cell of Cajal hyperplasia Motility disorders related to Cajal cell lesions
Neuroendocrine tumours	Carcinoid tumours at any gastrointestinal location Periampullary somatostatinoma Rarely, insulinoma and gastrinoma
Miscellaneous neoplasms and lesions	Adenocarcinoma at different gastrointestinal sites Vasculopathy

*NF1* neurofibromatosis type1, *GI* gastrointestinal tract, *GIST(s)* gastrointestinal stromal tumour(s), *MPNST* malignant peripheral nerve sheath tumor

## **Gastrointestinal tract lesions associated with NF1**

Gastrointestinal tract lesions are not uncommon in NF1 [6] and are reported in 10 to 25% of all cases [12, 14]. Involvement in NF1 patients almost always affects the upper gastrointestinal tract and includes tumours, vasculopathy and bleeding, pseudo­obstruction and protein­losing enteropathy [15]. Those gastrointestinal manifestations have delayed appearances compared with the cutaneous manifestations of the disease, occurring during midlife or later [6].

Gastrointestinal lesion occurrences in NF1 patients are mostly clinically occult [12]. Clinical manifestations are variable and depend on the location and extent of mucosal involvement, and symptomatic tumours are most common in the stomach and jejunum [12].

Mucosal involvement may lead to occult or profound gastrointestinal bleeding. Intestinal obstruction from intussusception or volvulus secondary to gastrointestinal neurofibromas may manifest themselves in nausea, vomiting and abdominal distension [12]. A list of the main abdominal lesions associated with NF1 is summarised in Table 2.

**Table 2:** Abdominal involvement of NF1

Abdominal involvement of NF1	
Gastrointestinal tract (GI tract)	Extra-intestinal
	Neurofibromas within The liver/mesentery/biliary ducts/retroperitoneum
*leiomyomas*	Mesenteric plexiform
*Adenocarcinomas*	Neurofibroma
*Neuroendocrine tumours*	Pheochromocytoma
GI tract vasculopathy	
GI tract bleeding	
Pseudo-obstruction	
Protein-losing enteropathy	

*GI* gastrointestinal

Most of the gastrointestinal neoplasms affect the upper gastrointestinal tract. Small intestines and particularly the jejunum and stomach are common sites of tumours [15, 16]. However, the oesophagus and colon are rarely involved [14].

Gastrointestinal tract neoplasms in NF-1 patients are more frequent than in the general population and occur in three main forms [6, 12, 17, 18]: hyperplasia of the submucosal and myenteric nerve plexuses and mucosal ganglioneuromatosis that leads to disordered gut motility; gastrointestinal stromal tumours (GIST) with varying degrees of neural or smooth muscle differentiation; distinctive glandular somatostatine-rich carcinoid in the periampullary region of the duodenum, which contains psammoma bodies and may be associated with pheochromocytoma.

Bakker and al [16] examined 61 reported cases of non-carcinoid gastrointestinal (GI) neoplasms in patients with NF-1. Neoplasms were located most often in the small intestine (72%). Neurofibromas (52%) were the most frequently diagnosed benign neoplasms followed by leiomyomas (13%), ganglioneurofibromas (9.8%) and gastrointestinal stomal tumour (GIST) (6.5%). Malignant tumour adenocarcinoma accounted for 23% in this review.

The coexistence of multiple tumours of different types is frequent in neurofibromatosis type 1 because of variable penetrance of the autosomal gene [17]. The patient may be subject to one or more tumours in synchronous or metachronous fashion [19].

Since there is no recommendation about modalities to explore these patients and because of the non-specific clinical presentation of GI lesion manifestations and limitations of endoscopic techniques, we suggest performing a CT enterography to detect these lesions. Indeed, with the advent of multidetector CT, improved contrast and spatial resolution, CT enterography may detect even the small lesions.

On the other hand, MR enterography, due to its high sensitivity for the detection of small bowel tumours, may be considered as an alternative to CT enterography particularly for younger patients or the follow-up.

## True neurogenic neoplasms of the gastrointestinal tract

Abdominal neurogenic neoplasms usually follow the distribution of the sympathetic ganglia along paraspinal areas or arise from the adrenal medulla or the organ of Zuckerkandl [20]. Other abdominal sites can be involved including the urinary bladder, bowel wall, abdominal wall and gallbladder [20].

Neurogenic neoplasms associated with NF1 include neurofibromas, ganglioneuromas and malignant peripheral nerve sheath tumours (MPNSTs).

## Neurofibromas

Neurofibroma is the hallmark lesion of NF1 [12]. Neurofibromas are benign nerve sheath tumours arising from Schwann cells, and the Auerbach plexus is the usual site of origin [15]. They may be unique or multiple with four subtypes, listed in Table 3: cutaneous, subcutaneous, nodular or diffuse plexiform and spinal [21].

**Table 3:** Different subtypes of neurofibromas

Intracutaneous neurofibromas	
• Late childhood or early adolescence	
• No malignant transformation	
Plexiform neurofibromas	
• 30–50%	
• Congenital	
• Possible malignant transformation	
Spinal neurofibromas	
• Solitary/multiple nerve roots	

Often multiple, neurofibromas of the gastrointestinal tract originate from the myenteric plexus. Histologically, they are composed of Schwann cells, fibroblasts and myxoid or mucinous matrix surrounded by collagenous tissue with mast cell infiltration and adipocytes, associated with cystic degeneration [12, 13, 21]. It may present as a solitary mass with smooth well-defined margins or a plexiform presentation [13, 21]. Malignant transformation is reported in between 5 and 15%, especially in patients of over 40 years of age [16].

**Fig. 2:**
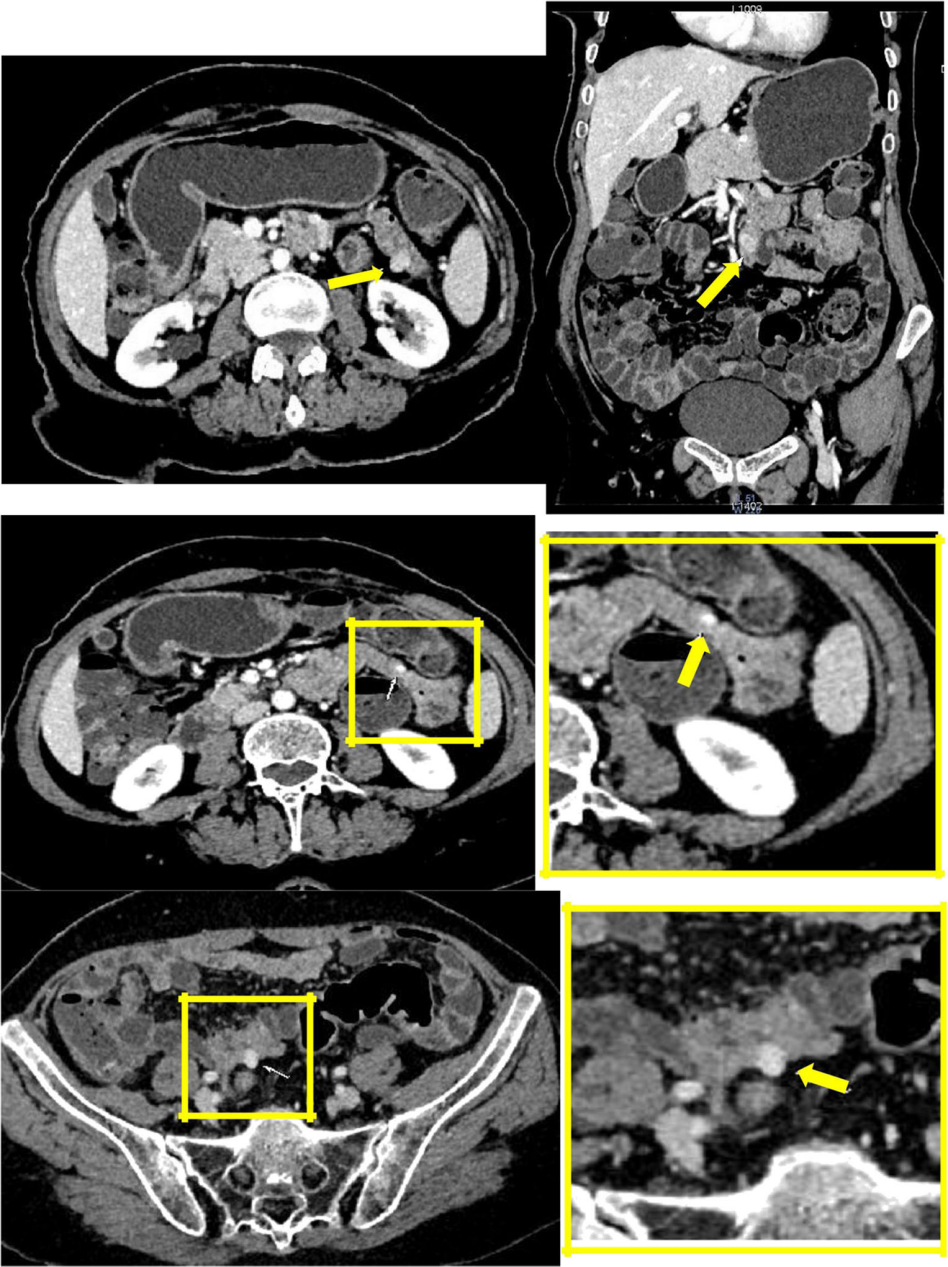
A 57-year-old female with systemic NF1. Patient presented vague abdominal pain. Dual-phase contrast-enhanced CT scan reveals multiples intestinal nodules highly enhanced by contrast (arrows)

Asymptomatic in 65%, GI tract neurofibromas are often incidentally detected during surgery for associated tumours [22]. Those tumours show up as thickening of the bowel wall or multiple nodules recognised at conventional barium examination as mural rigidity, external mass effect or scalloping of the mucosa [22]. On CT scan, they appear as homogeneously hypoattenuating round or tubular masses (Fig. 2). This characteristic reflects components of myxoid stroma, Schwann cells, adipocytes, and cystic degeneration [21]. On MRI, they present low signal intensity on T1-weighted images and variable signal on T2-weighted images with high signal intensity of cystic or myxoid areas and low intensity signal of the collagenous and fibrotic tissue that enhance after gadolinium administration [22].

Extra-peritoneal neurofibromas are often single and can be localised everywhere in the retroperitoneal space, mainly in the paraspinal position [12].

Plexiform neurofibromas are variants of neurofibromas that involve a plexus of nerves or multiple fascicles in a medium- to large-sized nerve within a larger nerve resulting macroscopically in “rope-like” or “bag-of-worms” aspects [12]. They are benign nerve sheath tumours usually affecting the head, neck, pelvis and extremities but can be found anywhere in the body [23]. They tend to grow to large sizes and may cause substantial disfigurement [12, 13]. Plexiform neurofibromas are seen exclusively in NF1; they are thought to be congenital and usually manifest early in life with an incidence of 15–30% [19, 22]. In the abdomen, they are most commonly found in the abdomino-pelvic wall and retro-peritoneum. Intra-hepatic infiltration is rare, comprising 2.3% of all plexiform neurofibromas of the abdomen and pelvis, and has been reported in fewer than 20 cases in the literature, 3 of which were in children [23].

**Fig. 3:**
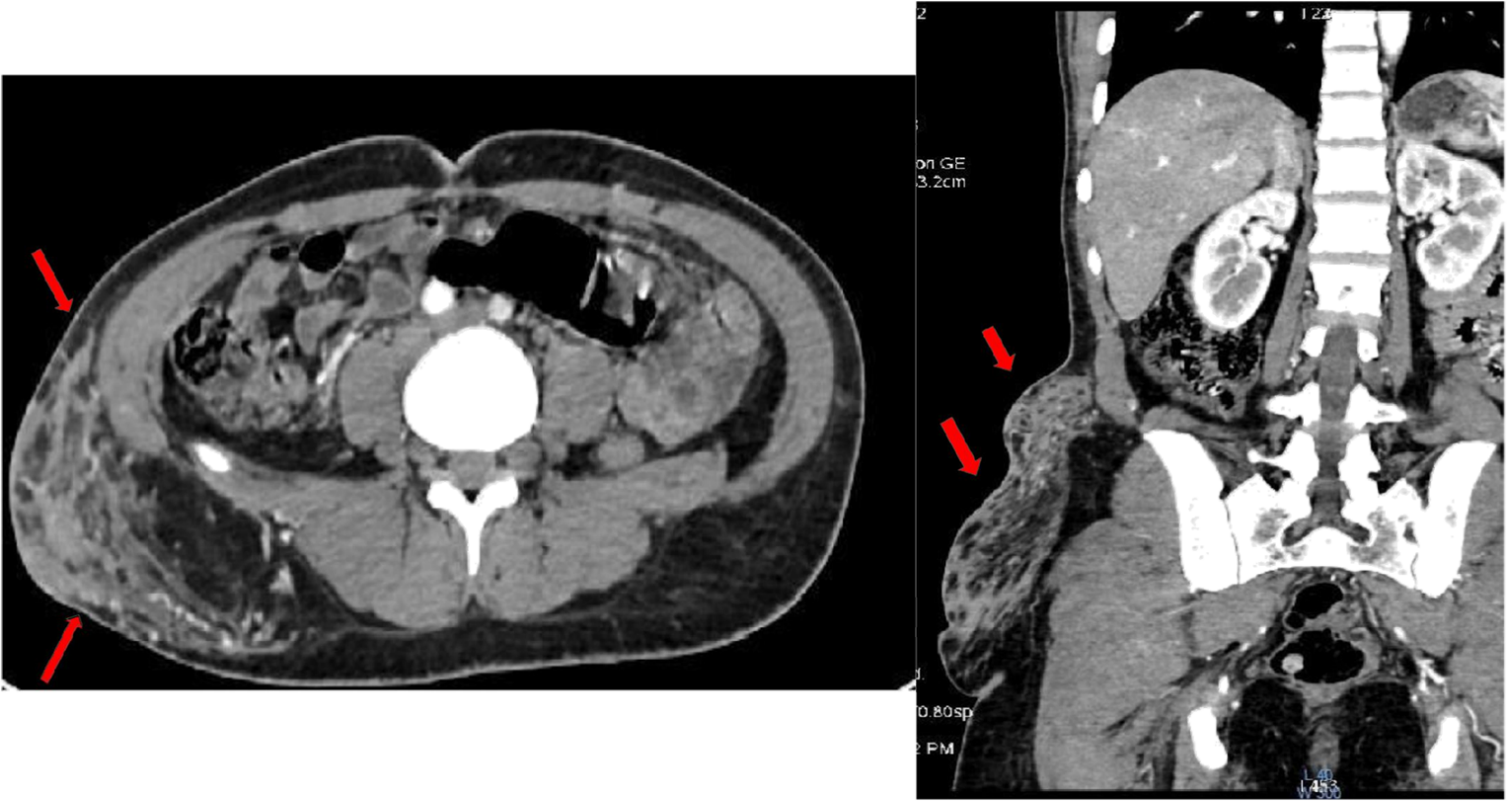
A 31-year-old female with a history of NF1 presenting a subcutaneous mass that feels like a “bag of worms”. CT scan showed an expansive heterogeneous image (arrows) located in the right lumbosacral external region (plexiform neurofibromas)

**Fig. 4:**
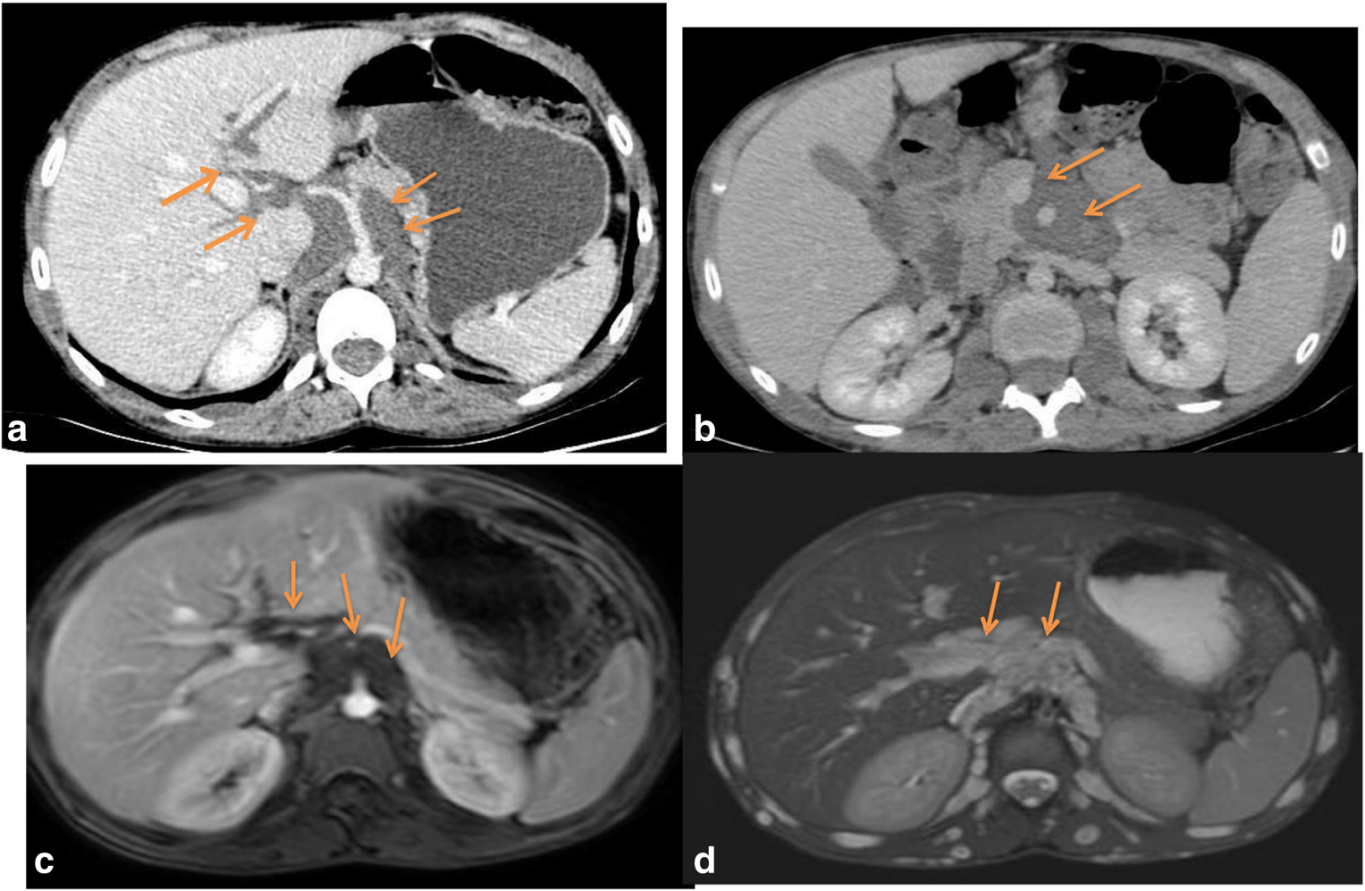
A 13-year-old boy with systemic NF1. Small bowel CT scan (**a**, **b**) and MRI (**c**, **d**) show a lobulated mass surrounding the retroperitoneal vessels extending from the left lateral aortic region to hepatic hilum (arrows)

On CT, plexiform neurofibromas appear as a multilobulated hypoattenuating tumour [21] (Fig. 3). They may rarely infiltrate the mesentery by extending along nerve pathways [21]. On MRI, plexiform neurofibromas appear with a characteristic “ring-like” pattern due to their fascicular architecture (Fig. 4).

Malignant transformation of neurofibromas to MPNSTs is possible [24]. It has been reported that most MPNSTs arise from preexisting plexiform neurofibromas and metastasise throughout the body [25].

## Malignant peripheral nerve sheath tumour

Previously referred to as “malignant schwannoma” or “neurofibrosarcoma”, MPNST is the most common malignant tumour associated with NF1 [24, 26]. It is a highly aggressive tumour located mainly in the paraspinal region of the abdomen, extremities, and head and neck region [12]. Approximately 50% of MPNSTs occur in patients with NF1, 10% are radiation induced, and the remainder of them occur sporadically [26]. Both sporadic and NF-1-associated variants of MPNST are quite uncommon in the gastrointestinal tract [6]. MPNSTs that arise in the abdomen and retro-peritoneum are often clinically silent, in contrast with other locations where the tumour is painful [12].

They may arise from preexistent neurofibromas in 10% [22], with the lifetime risk in patients with NF1 ranging from 4 to 14% [12], but it can also occur in the general population [24]. Ducatman et al. [27] reported a coexisting benign neurofibroma in 81%, which suggests an origin from a preexisting tumour.

Recent studies have discovered an association between microdeletions and higher risk of developing MPNST. Multiple studies reported MPNST associated with NF1 to present at younger age of onset (mean age of 26 years compared with 35 years in the general population), greater tumour size and higher disease stage [12, 26, 28]. The prognosis, however poor, remains similar in both patient with NF1 and the general population [12]. In fact, the prognosis depends on the tumour size and location, resection margin, adjuvant chemotherapy, distant metastasis, stage and site [28].

On imaging, MPNT appears as a large aggressive tumour with heterogeneous appearance in both CT attenuation and MR signal intensity related to the presence of necrosis [12]. The enhancement pattern is typically heterogeneous with irregular and infiltrative borders. MPNST may invade adjacent organs or even destroy adjacent vertebrae or pelvic bones [12]. Distinction from coexisting plexiform neurofibromas is difficult on cross-sectional imaging because both have similar imaging features, including bone erosion and heterogeneity [26].

The tumour may metastasise in 40–60% within 1 year [12]. Like in most sarcomas, the lung is the most common site for metastatic disease followed by the liver, adrenal gland, brain, bone and lymph nodes [26].

### Ganglioneuromas

This is a benign tumour of sympathetic ganglion cell origin composed of mature Schwann cells, ganglion cells and nerve fibres associated with an abundant collagenous stroma [20]. It can occur anywhere along the paravertebral sympathetic plexus or in the adrenal gland. Its occurrence in the GI tract is rare and may have three types of presentations [12, 20]: focal polypoid (ganglioneuromas) multifocal polyps (ganglioneuromatous polyposis) or a diffuse infiltrating lesion (ganglioneuromatosis), which is more frequent in the rectum than the colon. The latter two patterns occur more commonly in the colon and rectum in patients with NF1 or with multiple endocrine neoplasia type 2b [12] . Ganglioneuromas are usually asymptomatic even if they are large. Polypoid type may be determined with GI bleeding or signs and symptoms of intestinal obstruction [12]. Ganglioneuromas may be hormonally active secreting catecholamines, vasoactive intestinal polypeptides or androgenic hormones, which may be responsible for symptoms as hypertension, diarrhoea and virilisation [20]. Ganglioneuromatosis may result in motility disturbance, which can be severe enough to lead to a Hirschsprung-like condition in children and chronic colonic pseudo-obstruction and megacolon in adults [12].

This tumour tends to partially or completely surround major blood vessels, with little or no compromise of the lumen [20]. In the GI tract, focal ganglioneuromas are generally small, sessile or pedunculated polyps, whereas diffuse ganglioneuromatosis produces diffuse or nodular intestinal wall thickening [12].

At unenhanced CT, the tumour appears homogeneous, with attenuation less than that of muscle. Gradually progressive enhancement is seen on enhanced CT with delayed accumulation of contrast. On MRI, the tumour appears as intermediate low signal intensity on T1-weighted images. The importance of myxoid stroma explains the low signal on T2-weighted images with curvilinear bands that give the tumour a whorled appearance. Calcifications are present in 20% [20].

## Gastrointestinal stromal tumour

Gastrointestinal stromal tumour (GIST) is a mesenchymal tumour that represents the most common gastrointestinal manifestation of NF-1. It is reported in 5–25% of NF1 patients and accounts for 1.5% of all GISTs [6, 29]. GISTs associated with NF1 syndrome seem to have a distinct phenotype compared with sporadic GISTs: they occur in younger patients (mean age, 49 years versus 56 in sporadic GISTs), are multiple in 60% or develop in multiples sites, are smaller in size with low mitotic activity, follow a benign clinical course and occur mostly in the duodenum or small intestine [6, 10]. They are usually clinically asymptomatic and detected as incidental findings. They lack GIST-specific mutations; therefore, they show a variable but generally incomplete response to the tyrosine kinase inhibitor imatinib treatment [6, 10].

Typically, a GIST is a well-defined and exophytic mass with a clear delineation from the mesentery. An intraluminal mass is far less common [11].

**Fig. 5:**
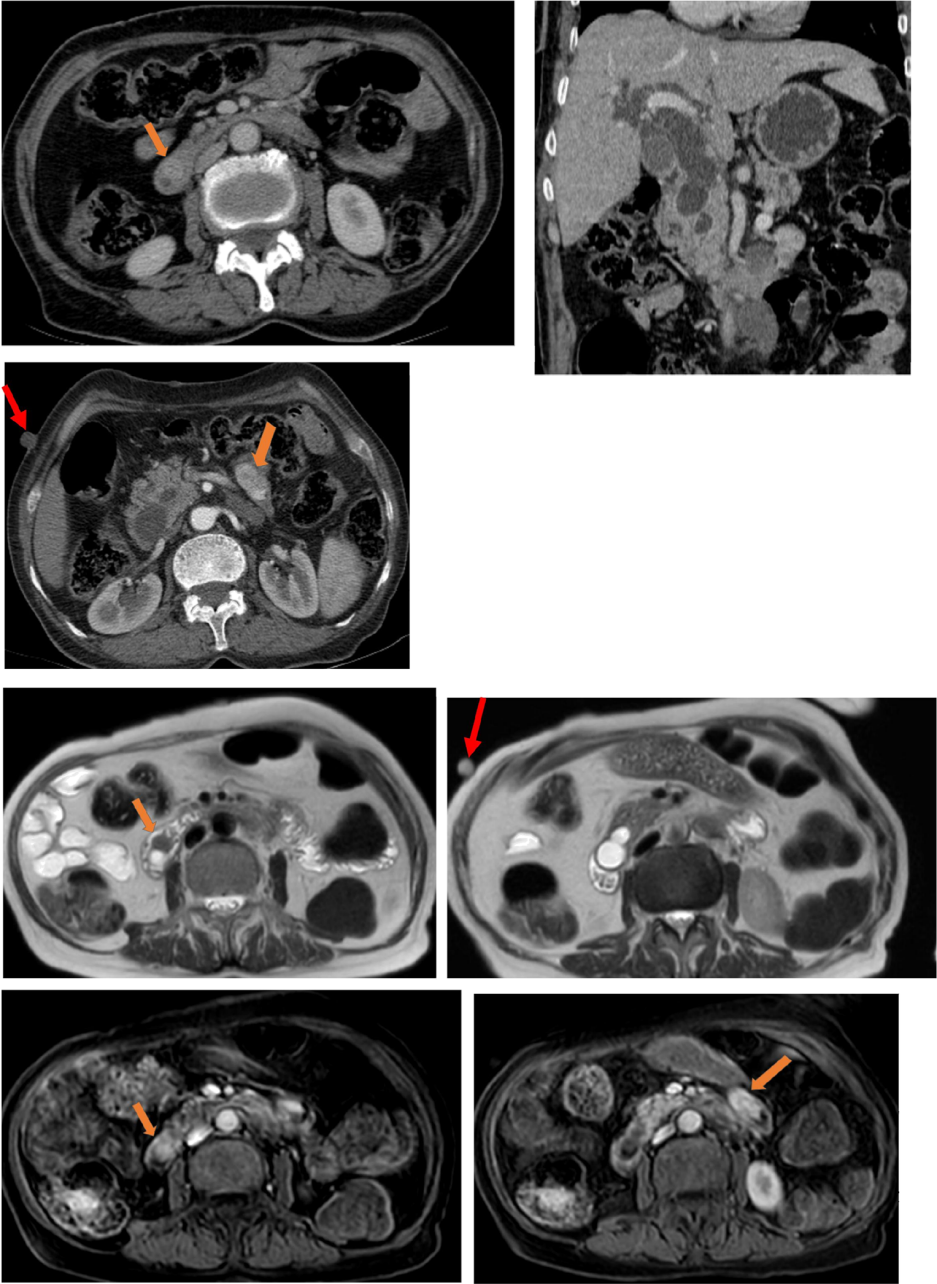
A 65-year-old female with no history of NF1, presenting a neurofibroma of the abdominal wall (red arrows) and two GI intraluminal masses: a peri-ampullary (orange arrows), which is responsible for the bicanalar duct dilatation (neuroendocrine tumour), and hyperenhanced duodenal lesion: GIST

CT shows typically enhancing masses, arising from the wall of a hollow viscus with an endoluminal or exophytic growth, with low attenuation from haemorrhage, necrosis or cyst formation (Fig. 5).

On MRI, the presence of necrosis, haemorrhagic and cystic change makes appearances variable. Typically, lesions show low signal intensity on T1-weighted images with usually peripheral enhancement in large lesions and high signal intensity of the solid component on T2-weighted images [12].

## Neuroendocrine tumours (NET) of the digestive system associated with NF1 (Figs. 5 and 6)

**Fig. 6:**
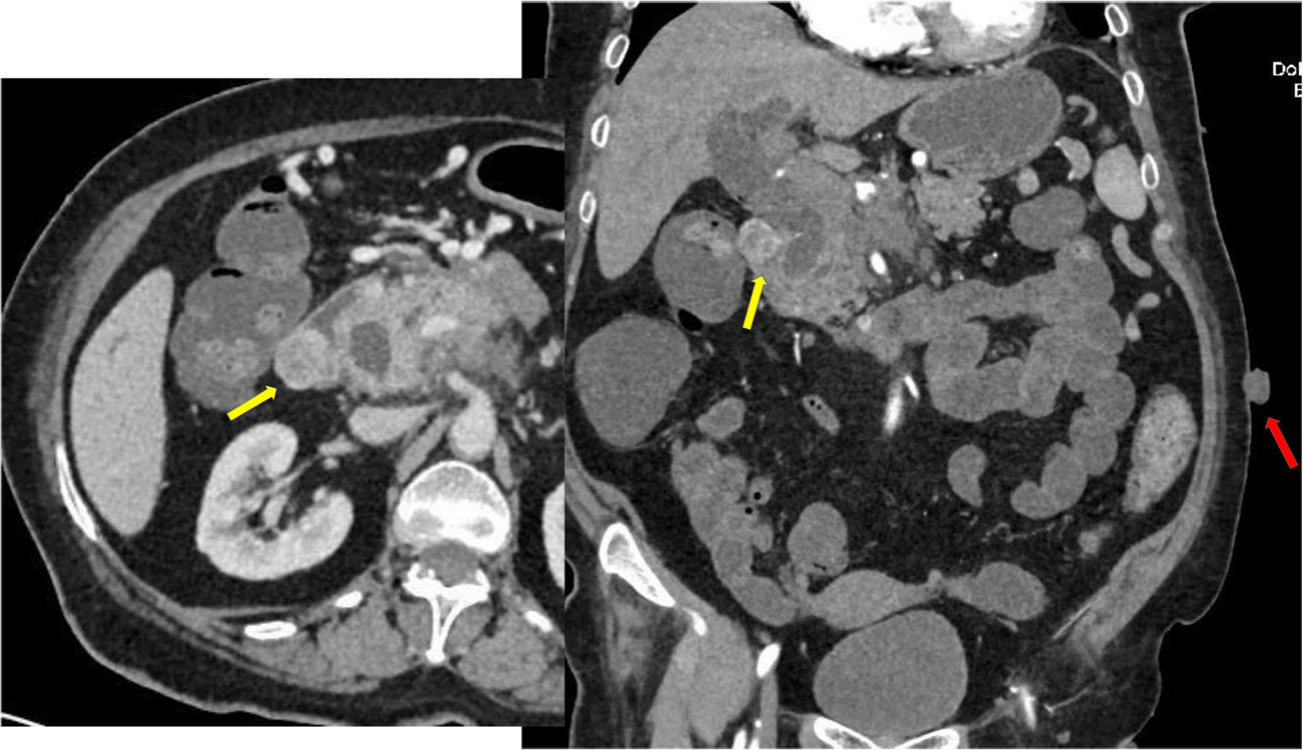
A 55-year-old female with systemic NF1 patient presenting a cholangitis. Dual-phase contrast-enhanced CT scan reveals a parietal pariampullary duodenal nodule highly enhanced by contrast (yellow arrows). Neurofibromas of the abdominal wall are also found on CT scan (red arrow)

NETs are reported in about 1% of individuals with NF1 [19]. NF1-related NETs are among other inherited syndromes such as multiple endocrine neoplasia type 1 syndrome or tuberous sclerosis [29]. Clinical symptoms are multiple and variable depending on the tumour size, compression and spread [19]. NF1-related NET may involve any part of the GI tract with special affinity for the duodenal and peri-ampullary region. Duodenal NET and NF1 has recently become a distinct syndrome as it is the most common site for NET associated with NF1 [29]. NETs may be functioning or non-functioning, depending on their hormonal activity (Fig. 6).

Somatostatinomas are the most common type of peri-ampullary NET in NF1 patients [6]. Carcinoid tumours and pheochromocytoma are also seen in NF1 patients with more frequency than in the general population and are frequently discovered at the same time in the same patient. The majority of duodenal carcinoids occurring in NF1 can be classified as somatostatinomas [12].

In NF1, somatostatinomas typically occur in the vicinity of the ampulla of Vater and less frequently in the pancreas. The majority of somatostatinomas involving the ampulla of Vater occur in the setting of NF1 [30]. They occur at younger age than in the general population (less than 50), are smaller in size because of earlier recognition related to local symptoms and well-differentiated with a low grade of malignancy (Table 4).

**Table 4:** Imaging characteristic features for gastrointestinal lesions associated with NF1

GI lesions type		General characteristic features	Imaging characteristic features for CT scan and MRI		Differential diagnosis
True neurogenic neoplasms	neurofibromas		-Homogenously hypo attenuating round or tubular masses	- Characteristically low signal intensity on T1-w - Heterogeneous high signal intensity on T2-w: • High T2 signal: pathological areas of cystic - degeneration or normal myxoid matrix • The low T2 signal: collagen and fibrous tissue - Areas of lowT2 signal enhance following - gadolinium administration	- Digestive schwannomas - Neurinomas - Neuroendocrine tumour - GIST
	Plexiform neurofibromas		-Nodules or infiltrating lesions – Extension from the root of the mesentery to the wall of the intestine – iso or hypoattenuating	Ring-like or septated pattern Rope-like gross appearance	- Pathognomonic
	Multiple nerve sheath tumour		Heterogeneous in CT attenuation and MR signal intensity (necrosis++) Enhancement pattern is typically heterogeneous Borders of the tumour are often irregular and infiltrative		- Plexiform neurfibroma
GIST		- Younger age - Multiple lesions - Asymptomatic - No overexpression of KIT or PDGFRA	- No particularity	- No particularity	–
Neuroendocrine tumours		- More frequent particularly in black population - Younger age of presentation - Same range of malignancy as general population - Always arise in or near the ampulla of Vater - Somatostatin secretion - Rarely symptomatic - Association with pheochromocytoma and duodenal carcinoids in NF1	- No particularity	- No particularity	–
GI tract vasculopathy		- Not before age 5O -Affects aorta and its main branches	-Arteriovenous malformations - Vascular stenosis and post-stenotic aneurysm -Stenosis usually involves multiples territories - Aneurysms do not occur in two different circulatory regions		Athrosclerosis
Adenocarcinoma Embryonal tumour		Association with NF1 controversed	- No particularity		No particularity

*T1‐w* T1 weighted image, *T2‐w* T2 weighted image, *KIT* tyrosine kinase receptor, *PDGFRA* platelet derived growth factor alpha, *NF1* neurofibromatosis type1, *GI* gastrointestinal tract, *GIST* gastrointestinal stromal tumour

There is a high incidence of psammoma bodies (psammomatous calcifications) in the duodenal lesions of patients with NF1, which may be helpful in establishing the diagnosis [31]. The majority of peri-ampullary somatostatinomas are non-functioning. Therefore, the somatostatin syndrome, including diabetes mellitus, steatorrhea and weight loss, is absent in NF1 individuals. Symptoms are the result of the mass effects: jaundice and non-specific abdominal pain. In NF1 individuals, these tumours very rarely metastasise.

The imaging features of a peri-ampullary mass in a patient with NF1 are clinically important because pancreatic and peri-ampullary adenocarcinoma are difficult to distinguish histologically from a peri-ampullary carcinoid. A peri-ampullary carcinoid presents as a focal intraluminal mass [12]. Gangliocytic paragangliomas of the duodenum and adenocarcinoma of the pancreas are the main differential diagnoses with a benign histological and clinical pattern.

Pheochromocytoma is more common in patients with NF1 than in the general population, especially when associated with GISTs. Pheochromocytomas are found in up to 20% of patients presenting hypertension [11, 12].NF1-associated pheochromocytoma presents as a solitary and unilateral encapsulated mass expanding from the adrenal medulla with no particular imaging features compared with the general population.

## Miscellaneous neoplasms and lesions

## Vasculopathy associated with NF1

**Fig. 7:**
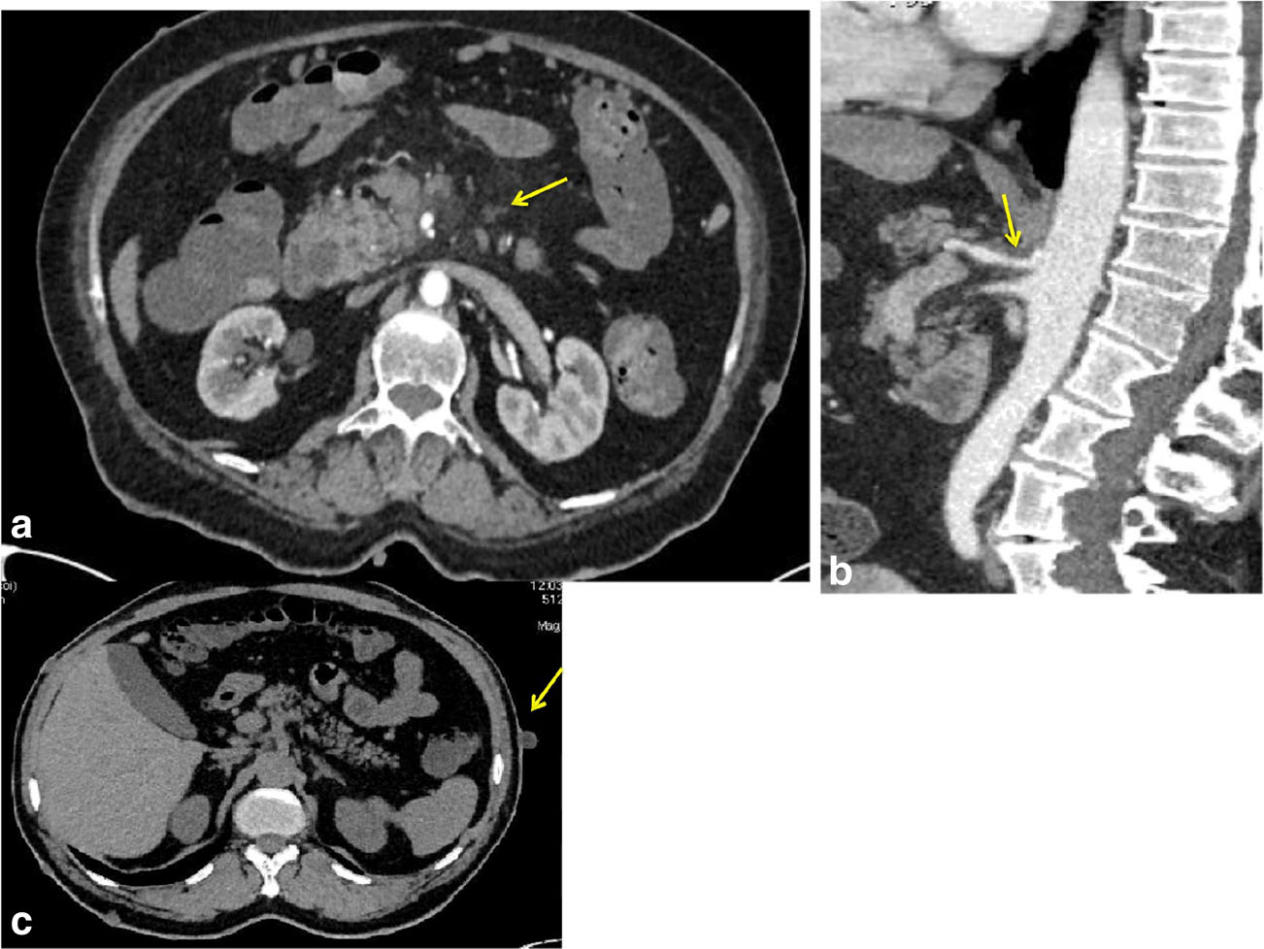
A 53-year-old male with a history of NF1. A small bowel CT scan showed no gastrointestinal tract involvement. **a**, **b** CT scan showed ostial stenosis of the coeliac trunk and superior mesenteric artery (arrows). **c** Multiple hypdense cutaneous nodules on both sides of the abdominal wall (neurofibromas)

Intrinsic lesions of arterial walls are important manifestations of NF1, occurring in 3.6%, and are the second cause of death in individuals with NF1. It affects mostly the aorta and its main branches [32]. The renal artery is the most common site of involvement resulting in renovascular hypertension. The NF1 protein, neurofibromin, is robustly expressed in endothelial cell vessels and smooth muscle cells [32] (Fig. 7).

NF1-related vasculopathy includes narrowed or ectatic vessels, vascular stenosis, aneurysm or arteriovenous malformations [33]. Arterial stenosis has been the most commonly reported lesion. Aneurysms are less common and are often post-stenotic [34]. Investigators have noted intimal thickening, thinning of the media and parietal dilatation in small vessels, which may result in fragile dysplastic vessels [33].

Oderich et al. [33] identified a predominance of aortic, renal and mesenteric lesions in NF1-related vasculopathy. Aneurysms or stenoses occur by age 50 years and are due to an underlying vasculopathy, whereas the older ones had degenerative atherosclerosis. Although atherosclerosis typically involves the origin or bifurcation of large arteries, NF-I vascular lesions are tapered and extended into the primary branches of the artery.

Stenosis usually involves multiples territories, whereas aneurysms do not occur in two different circulatory regions. The real incidence of coeliac and superior mesenteric artery vasculopathy is unknown because of lack of diagnosis. Only nine cases of superior mesenteric artery aneurysm have been reported in association with NF1 [33, 34].

## Adenocarcinomas

Adenocarcinomas involving the whole gastrointestinal tract have been detected in patients with NF1 but the association is unclear. Indeed, the occurrence of adenocarcinomas in the colon, oesophagus, stomach, biliary tract and pancreas with NF1 may be incidental [7]. Adenocarcinomas of the small bowel instead seem to de associated to NF1 given the increased incidence in NF1 patients, particularly in the periampullary site. However, this association is a controversial topic because of the well-established association between carcinoids and NF1 and the difficulty in distinguishing between adenocarcinomas and carcinoids histopathologically [12].

## Embryonal tumours

Three embryonal tumours have a reported association with NF1: rhabdomyosarcoma, neuroblastoma and Wilms’ tumours. Although the association between NF1 and rhabdomyosarcoma is evident because of the accepted role of the NF1 gene in the differentiation of muscular cells, which explains a common pathogenetic mechanism, associations between neurofibromatosis and Wilms’ tumour or neuroblastoma are reported, but are not confirmed from a genetic and molecular point of view [7, 12].

## Conclusion

Patients with NF1 are predisposed to both benign and malignant tumours that may have neurogenic or non-neurogenic origins. These lesions are significantly under-recognised in clinical practice. Early diagnosis of these gastrointestinal manifestations is very important because of the risk of malignancy and organic or haemorrhagic-obstructive complications. The unforeseeable course of NF1 and the lack of a resolute cure lead to the necessity of a strict follow-up managed by an expert multidisciplinary team [13]. Surgical removal, when technically feasible, prevents local infiltration and malignant transformation [22].

## Abbreviations

CT: Computed tomography
GI: Gastrointestinal
GIST(s): Gastrointestinal stromal tumour(s )
MPNST(s): Malignant peripheral nerve sheath tumour(s)
MR: Magnetic resonance
NET: Neuroendocrine tumours
NF1: Neurofibromatosis type 1
